# Concussion May Result in New-Onset Bipolar Disorder: A Case Report

**DOI:** 10.7759/cureus.64731

**Published:** 2024-07-17

**Authors:** Kent W Sabatose, Nichole Cufino, Wendy Hahn, Murat Ibatullin

**Affiliations:** 1 Osteopathic Medicine, Lake Erie College of Osteopathic Medicine, Bradenton, USA; 2 Behavioral Health, Lake Erie College of Osteopathic Medicine, Bradenton, USA; 3 Medical Imaging, Lake Erie College of Osteopathic Medicine, Bradenton, USA

**Keywords:** white matter injury and traumatic brain injury, post traumatic brain injury, diathesis stress model, adult concussion recovery, concussion applications, brain and bipolar disorder, bipolar disorders, diffuse axonal injury and traumatic brain injury

## Abstract

Emotional dysregulation following a concussion is well established. New onset of major psychiatric diseases such as bipolar disorder (BPD) post-concussion has not been investigated. BPD typically presents with an initial depressive episode followed by mania and concurrent depressive and manic states. Multiple theories explaining the etiology of BPD exist, including the diathesis-stress model (DiSM), though an accepted theory is not established. In this case study, medical records of a 50-year-old ambidextrous male with co-morbid attention deficit hyperactivity disorder (ADHD) inattentive type, obsessive-compulsive disorder (OCD), and a family history of BPD suffered a motor vehicle collision (MVC) resulting in a grade II concussion. New onset BPD was diagnosed one-year after a concussion following an involuntary admission and led to the patient self-terminating his medications and suffering a hypertensive crisis and aortic dissection, and stroke. One year later, the patient was again involuntarily admitted for a suicide attempt. Bipolar disorder persisted unchanged indefinitely. This case study may serve as a real-world example of the DiSM in the etiology of BPD post-concussion. We aim to highlight the importance of early identification of risk factors for progression to psychiatric conditions following concussion.

## Introduction

The etiology of psychiatric conditions such as bipolar disorder and schizophrenia remains under investigation. Though likely multidimensional, one explanation may be the diathesis-stress model (DiSM), describing the onset of psychiatric disease resulting from a significant life stressor in a genetically susceptible person leading to the phenotypic expression of a previously silent condition [1]. Traumatic brain injury may serve as a well-suited life stressor capable of inducing such a reaction in genetically susceptible individuals. In this case report, we describe a genetically susceptible individual who developed new-onset bipolar disorder with mixed presentation following a traumatic brain injury resulting in significant medical consequences. 

This case report aims to highlight the importance of early identification of pre-existing risk factors in patients suffering traumatic brain injury. Preventative measures or frequent monitoring may be implemented for those who are at greater risk of psychological deterioration following the inflammatory brain response to brain injury. 

## Case presentation

**Figure 1:**
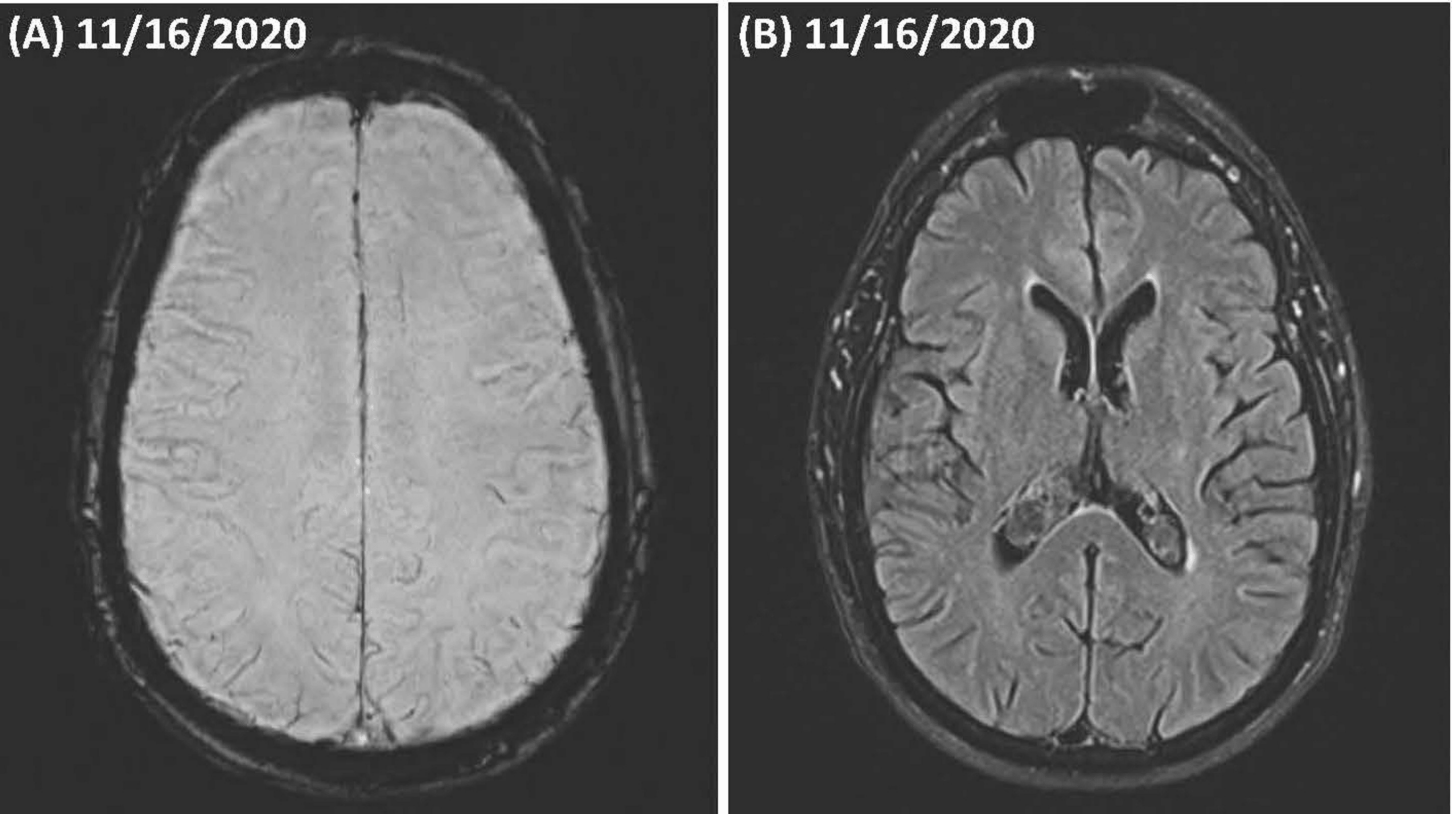
Brain MRI post-concussion (A) Susceptibility-weighted imaging (SWI) sequence showing no hemosiderin deposition. (B) Fluid-attenuated inversion recovery (FLAIR) sequence imaging showing mild hyperintensities of frontal and parietal periventricular white matter.

A 50-year-old ambidextrous male presented to an outpatient neurorehabilitation clinic due to a history of dizziness, imbalance, and changes in personality, behavior, and mood following a motor vehicle accident (MVA) 20 months prior to examination. Past medical history included obsessive-compulsive disorder, attention deficit hyperactivity disorder, and hypertension. The patient’s family medical history included a sister and brother diagnosed with bipolar disorder and a mother with a history of stroke. The patient reported striking his forehead on the steering wheel, resulting in hematoma, a feeling of dizziness, and tinnitus without loss of consciousness, leading to a grade II concussion diagnosis.

(Figure 1) shows a brain MRI, two months post-MVC showing negative susceptibility weighted imaging (SWI), mildly increased fluid-attenuated inversion recovery (FLAIR) signal in the frontal and parietal periventricular regions, and (not shown) abnormal diffusion tensor imaging (DTI) with cerebral fractional anisotropy score in trauma (C-FAST) score of 4 consistent with grade II concussion with diffuse axonal injury. Post-concussion symptoms included dizziness and balance disturbance, which was managed with vestibular therapy. Worsening mood disturbance and personality changes were noted over the next year by the patient’s caregiver.

**Figure 2:**
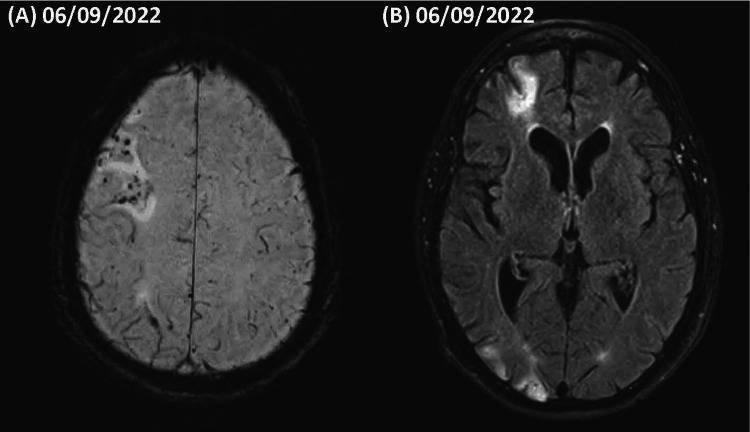
Brain MRI post-stroke (A) Susceptibility weighted sequence showing diffuse hemosiderin deposition and clustering in the right frontal lobe. (B) FLAIR sequence post aortic dissection with stroke. Note increased signal in the right frontal and occipital lobes.

One year following the MVA, the patient experienced increased irritability, anxiety, paranoia, rapid speech, hypersexuality, and aggression, requiring involuntary admission into a psychiatric center where a diagnosis of BPD with mixed presentation was established. He was treated with and discharged on oxcarbazepine, risperidone, and trazodone. Post-discharge, the patient lost his business of 36 years, became homeless, and stopped his medications. Two months after discharge, the patient was admitted to the hospital due to a syncopal episode and hypertensive crisis resulting in aortic dissection and stroke. (Figure 2) shows widespread involvement of embolic stroke following aortic dissection, revealing increased FLAIR signal in the right frontal, parietal, and occipital lobes with frontal encephalomalacia. Scattered SWI blooming clustering in areas of increased FLAIR signal can be appreciated. Following the stroke, the patient experienced dysarthria and visual disturbance, complicating the vestibular and psychiatric symptoms.

Approximately one year later, the patient was involuntarily admitted a second time to a psychiatric center following a suicide attempt with severe depressive symptoms consistent with acute bipolar depression. He was stabilized and discharged on medications noted above with the addition of sertraline, trazodone, and lorazepam. The patient continued to experience BPD symptoms until he was lost to follow-up.

## Discussion

The American Congress of Rehabilitation Medicine diagnostic criteria for mild traumatic brain injury (mTBI) requires one or more criteria, including clinical, laboratory, and neuroimaging signs/evidence following a biomechanically plausible mechanism of injury [2,3]. Secondary to the physical injury induced by the initial force, both an oxidative stress response and microgliosis reaction occur, which lasts at least 7 weeks [4,5]. As a result, axonal injury and neuronal cell death may occur months following TBI, leading to both persisting and novel symptoms in the post-concussional phase. In our case report, our patient had both pre-existing and TBI-induced neuropsychiatric conditions serving as significant risk factors for a poor prognosis of mTBI symptoms leading to post-concussional syndrome.

According to the DSM-5, BPD is a mood disorder that encompasses BPD type I, type II, and cyclothymia, all based on the absence or presence of manic and major depressive episodes [6]. Bipolar I disorder is characterized by at least one manic episode being present for more than one week or severe enough that it leads to hospitalization. If there was a major depressive episode, it would have to be present for more than two weeks [6]. Interestingly, the onset of BPD typically occurs at about 32 years old, yet this case study presented manic symptoms at 50 years old. Additionally, the onset of BPD can occur comorbidly in the elderly with organic medical conditions such as dementia or medication-related adverse events, also not fitting our patient’s demographics [6].

Although BPD has been ranked as the sixth out of the ten most common causes of medical disability, there is still insufficient research on the etiology of the disorder [7]. Some current biological theories of the etiologies of BPD are disruptions in emotion and executive functioning, the hypothalamic-pituitary-adrenal (HPA) axis, voltage-gated calcium channels, and increased prevalence of immunologic markers in adolescence. Other psychosocial theories suggest that the presence of a manic episode can be a defense against a depressive episode or from an inability to cope with a developmental tragedy [6]. Based on this patient’s presentation of BPD after the motor vehicle collision, we hypothesize that another possible etiology for BPD is the diathesis-stress model. This model describes individuals with a genetic disposition for a psychiatric disorder that can remain undetected until a psychosocial stressor occurs and presents this illness transiently or permanently. This case study presented with an onset of BPD following his motor vehicle collision, which mirrors the tragedy or stressful life event leading to the onset of psychiatric illness. Parmigiani et al. found that perceived stress contributed to the onset and increase of psychotic symptoms in a sample of individuals with BPD or schizophrenia [8]. These findings align well with our hypothesis and can provide an additional factor to consider when managing the care of patients following a traumatic life stressor. 

In a national survey conducted in 2022, 48% of patients with no history of mental illness received a mental illness diagnosis within three-years of TBI [9]. Our patient presented a progression toward a diagnosis of BPD over the course of one year. Pre-existing neuropsychiatric conditions, including Alzheimer's disease, Parkinson’s disease, mild cognitive impairment, depression, mixed affective disorders, and BPD, have a known correlation with prior TBI. This correlation was found strongest between mild cognitive impairment, depression, and BPD [10]. Despite the known correlation between TBI and neuropsychiatric illness, factors that increase susceptibility post-TBI to neuropsychiatric disorders such as BPD have been poorly defined and require further investigation, including if multiple TBIs alter the risk for subsequent neuropsychiatric disease [10]. 

Mortensen et al. showed an increased risk of a history of TBI in patients admitted for BPD [11]. Alternatively, patients with a history of neuropsychiatric illness have an estimated five times greater risk of developing post-concussion syndrome compared to the general population [12]. Therefore, a bidirectional relationship likely exists, creating a positive feedback loop that increases the incidence of both TBI and BPD. The relationship between TBI and BPD requires further investigation due to the high prevalence, especially among adolescent populations.

There are limitations to this study. The first limitation is the retrospective nature of the report. Some of the data was also obtained through documentation from multiple physicians at multiple facilities. Finally, long-term follow-up with the patient was incomplete. Future analysis with prospective longitudinal studies of patients following TBI exhibiting risk factors for BPD should be performed.

## Conclusions

The presented case may serve as a real-world example of the diathesis-stress model (DiSM) with traumatic brain injury as a stimulus, culminating in a new diagnosis of Bipolar disorder (BPD). Undiagnosed or late diagnosis of new-onset psychiatric conditions poses a significant health risk to patients, as displayed in this case presentation. Psychiatric evaluation and screening for risk factors of psychiatric conditions following traumatic brain injury should not be overlooked to provide early diagnosis and management.
